# Long-term Survival Outcomes of Laparoscopic Versus Open Gastrectomy for Gastric Cancer: A Systematic Review and Meta-analysis

**DOI:** 10.1097/MD.0000000000000454

**Published:** 2015-01-30

**Authors:** Xin-Zu Chen, Lei Wen, Yuan-Yi Rui, Chao-Xu Liu, Qing-Chuan Zhao, Zong-Guang Zhou, Jian-Kun Hu

**Affiliations:** From the Department of Gastrointestinal Surgery, West China Hospital, Sichuan University, China (XZC, YYR, ZGZ, JKH); Department of Surgery, Xijing Hospital of Digestive Diseases, The Fourth Military Medical University, China (LW, CXL, QCZ).

## Abstract

Many meta-analyses have confirmed the technical feasibility and favorable short-term surgical outcomes of laparoscopic gastrectomy (LG) for gastric cancer patients, but the long-term survival outcome of LG remains controversial compared with open gastrectomy (OG).

This study aimed to compare the 5-year overall survival (OS), recurrence, and gastric cancer–related death of LG with OG among gastric cancer patients.

PubMed was searched to February 2014.

The resectable gastric cancer patients who underwent curative LG or OG were eligible. The studies that compared 5-year OS, recurrence, or gastric cancer–related death in the LG and OG groups were included.

A meta-analysis, meta-regression, sensitivity analysis, subgroup analysis, and stage-specific analysis were performed to estimate the survival outcome between the two groups and identify the potential confounders. Quality assessment was based on a tailored comparability scoring system.

Twenty-three studies with 7336 patients were included. The score of comparability between two groups and the extent of lymphadenectomy were two independent confounders. Based on the well-balanced studies, the 5-year OS (OR = 1.07, 95% CI 0.90–1.28, *P* = 0.45), recurrence (OR = 0.83, 95% CI 0.68–1.02, *P* = 0.08), and gastric cancer–related death (OR = 0.86, 95% CI 0.65–1.13, *P* = 0.28) rates were comparable in LG and OG. Several subsets such as the publication year, study region, sample size, gastrectomy pattern, extent of lymphadenectomy, number of nodes harvested, and proportion of T1–2 or N0–1 did not influence the estimates, if they were well balanced. Particularly, the stage-specific estimates obtained comparable results between the two groups.

Randomized controlled trials comparing LG with OG remain sparse to assess their long-term survival outcomes.

The major contributions of this systematic review compared with other meta-analyses are a comprehensive collection of available long-term survival outcomes within a much larger number of observations and a more precise consideration of confounders. Current knowledge indicates that the long-term survival outcome of laparoscopic gastric cancer surgery is comparable to that of open surgery among early or advanced stage gastric cancer patients, and LG is acceptable with regard to oncologic safety.

## Abstract 2

Supplemental Digital Content is available in the text

## INTRODUCTION

Gastric cancer is the fourth most common cancer and the second most common cause of cancer death worldwide.^1,2^ Radical gastrectomy with lymphadenectomy is the essential curative approach for resectable gastric cancer patients.^3,4^ Laparoscopy-assisted distal gastrectomy for early gastric cancer was first introduced in 1991.^5^ During the latest two decades, laparoscopic gastric cancer surgery has become increasingly common in eastern Asia.^6–8^ In Western countries, laparoscopic gastrectomy (LG) for gastric cancer has received much attention.^9,10^

In the early period of the technique, LG was generally performed only for resection of early gastric cancers. A meta-analysis of randomized controlled trials concluded that laparoscopy-assisted gastrectomy (LAG) had a short-term advantage in the early recovery of gastric cancer patients such as by decreasing intraoperative blood loss and postoperative early morbidity.^11^ A recent report of a large Japanese nationwide cohort found similar beneficial results by laparoscopic distal gastrectomy among early gastric cancer patients.^12^ A growing number of reports has demonstrated the technical feasibility and safety of LG for locally advanced gastric cancer.^13,14^

With a high mortality-to-incidence ratio, the management of gastric cancer is challenging.^2^ The long-term survival effectiveness of LG is still pending considering pneumoperitoneum carbon dioxide, intra-abdominal hyperpressure, greater procedural complexity, a longer operation time, and a lower lymph node harvest.^11,15^ Concerning the oncological aspects, the application of LG for gastric cancer has been questioned because of early reports of port-site metastases.^16^ Reduced lymph node retrieval might violate the curability of a potentially radical resection.^17^ Pneumoperitoneum carbon dioxide and a prolonged operation time might impair the immune defense against metastasis and peritoneal seeding.^18,19^

The long-term survival of cancer patients is a key measure of the effectiveness of health care systems.^20^ This systematic review comprehensively searched available studies and performed meta-analyses to compare the 5-year survival outcomes of LG with those of conventional open gastrectomy (OG).

## METHODS

### Search Strategy

A comprehensive PubMed search from January 1990 to February 2014 was performed using the following strings: ”Stomach Neoplasms”[Mesh] AND ”Laparoscopy”[Mesh] AND (”1990”[PDAT]: ”2014”[PDAT]) AND ”humans”[MeSH Terms] AND English[lang]. Reference lists of systematic reviews or meta-analyses were additionally checked to identify potential eligible studies. The language of all of the publications was limited to English.

### Study Eligibility

The eligible studies were selected according to the following criteria: (1) randomized or nonrandomized comparative studies were considered; (2) the patients included were diagnosed with gastric cancer; (3) early or locally advanced candidates were acceptable; (4) there were no limitations for race, age, or gender; (5) the staging system was based on the individual reports; (6) the patients in the LG and OG groups were compared; (7) the laparoscopic procedures mainly included LAG, and additionally totally laparoscopic gastrectomy (TLG) and hand-assisted laparoscopic gastrectomy (HALG) were also considered; (8) any extent of lymphadenectomy from D1 to D2+ was acceptable; (9) in the LG and OG groups, the range of follow-up length should cover 60 months; (10) all the potentially eligible studies should report at least one of the primary outcome measures, including the 5-year overall survival (OS), tumor recurrence, and gastric cancer–related death rates; and (11) the numbers of events could be extracted from the original reports.

### Selection and Data Extraction

The procedures were performed in a peer-review manner by two independent reviewers. The general information that was extracted included the publication year, sample size, study design, general patient characteristics, and intervention details. The dichotomous data for the outcome measures mentioned above were extracted, including the total number of participants and events for each group. The number of events was calculated by the actual reported percentages, if possible. If the OS survival curves were presented, the number of individuals at risk was calculated by extracting the values at each inflexion. The recurrence data could be reported directly in the full text or calculated from the 5-year disease-free survival rate.

### Quality Assessment

The quality of each included study was assessed by two approaches. First, the comparability (comparable, unclear, or not comparable) of 13 relevant items, including the tumor site, tumor size, histological differentiation, stage, patient age, patient sex, proportion of distal gastrectomy, proportion of D2 lymphadenectomy, number of nodes harvested, postoperative mortality, adjuvant chemotherapy received, length of follow-up, and percentage of loss. These 13 items were considered to be associated with the survival outcomes as potential confounders. Second, a cumulative quality score was calculated based on the above 13 items. If an item were comparable between the two groups, a score of “”0”’ was given. If the balance of any item were unclear or not comparable, scores of “1”’ or “‘3,” respectively, were given. A higher cumulative comparability score indicated a lower quality study. The reason why the scale was nonlinear is because of linear scale (0, 1, and 2) unable to both underline the incomparability and result in an enough wide range of accumulative scores to determine an efficient cutoff to exclude heterogeneous studies (data not shown).

### Statistics

The STATA 12.0 statistical software was used for the synthesis and analysis. (1) The meta-analyses were performed initially using a fixed effects model. An odds ratio (OR) with a 95% confidence interval (CI) was calculated for the dichotomous data. The Mantel-Haenszel method was used to test the significance of the dichotomous data in a meta-analysis. Forest plots showed the results of the meta-analyses. A *P* value of less than 0.05 was considered statistically significant. (2) The between-study statistical heterogeneity was tested by a standard chi-square test, and a random effects model was used for a *P* value of less than 0.1. (3) Begg test and Egger test were performed to test for a publication bias, and the results were presented in a funnel plot. (4) To select any potential confounders, meta-regressions were performed in four models, including the study feature, tumor comparability, operation comparability, and postoperative comparability. A *P* value of less than 0.05 was considered to function as a confounder. (5) A sensitivity analysis was performed to determine an optimal cutoff of the quality scores and distinguish the quality of an individual study as good or poor. The meta-analyses were resynthesized from the low to high scoring subsets. A scatter plot with quadratic fit and a 95% CI area was drawn to observe the correlation between the scores and the ORs. (6) In addition, the subgroup analyses of the well-qualified studies and stage-specific analyses were performed to identify a contribution by an individual factor.

### Ethics, standards of reporting, data availability

This systematic review was not submitted to any biomedical ethical committee for approval, and meanwhile no consent was required from the analyzed individuals. This systematic review was performed and reported according to the PRISMA standard. All the data are fully available from the published papers.

## RESULTS

### Literature

**FIGURE 1:**
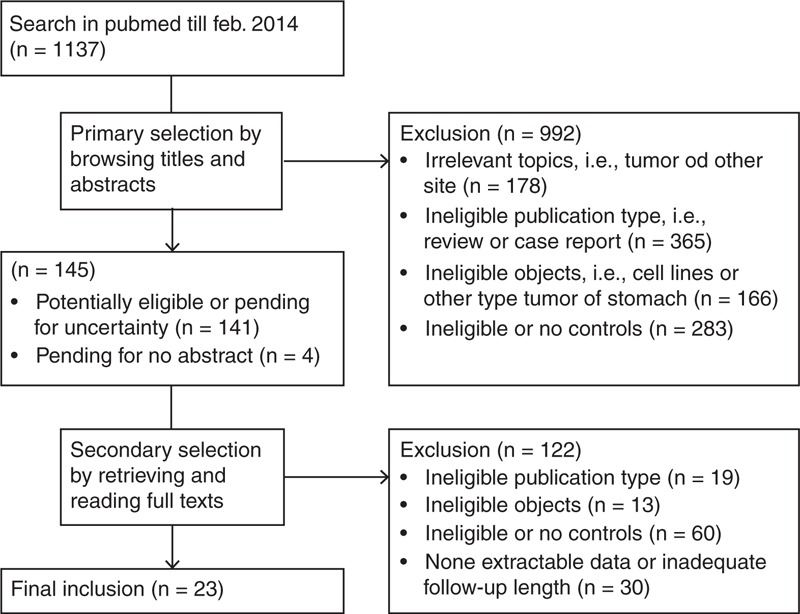
Literature search and selection procedures.

The procedures of the literature search and selection are shown in Figure 1. A total of 23 studies were selected from 1137 citations. A total of 7336 (3368 vs 3968) subjects were included in the LG and OG groups. There were two randomized controlled trials, whereas 21 studies were case-control studies. The studies were regionally distributed as follows: four from Italy, five from Japan, nine from Korea, and five from China. Twenty studies used the LAG technique, two used TLG, and one used TLG and HALG. The OS results were extracted from 22 studies, the recurrence results from 17 studies, and the gastric cancer–related death results from nine studies. The details of the studies including the quality assessment results are shown in Supplementary Table 1 (http://links.lww.com/MD/A184).^21–43^ The reasons for the exclusion of 10 comparative studies that reported survival outcomes are shown in Supplementary Table 2 (http://links.lww.com/MD/A185).^44–53^

### Five-year overall survival

#### All-pooled meta-analysis

In an all-pooled manner, 22 studies that reported the 5-year OS were synthesized in a meta-analysis (Supplementary Figure 1 http://links.lww.com/MD/A182). The LG group presented a better OS outcome than did the OG group in this initial analysis by random effect model due to significant between-study heterogeneity (I-squared = 82.7%, *P* < 0.01). No publication bias influenced the meta-analysis (Supplementary Figure 2 http://links.lww.com/MD/A183). Meta-regression found the cumulative comparability score and the comparability of D2 lymphadenectomy were independent confounders (Supplementary Table 3 http://links.lww.com/MD/A186). These two confounders were tested in subsequent sensitivity analyses and subgroup analyses.

#### Sensitivity analyses

**FIGURE 2:**
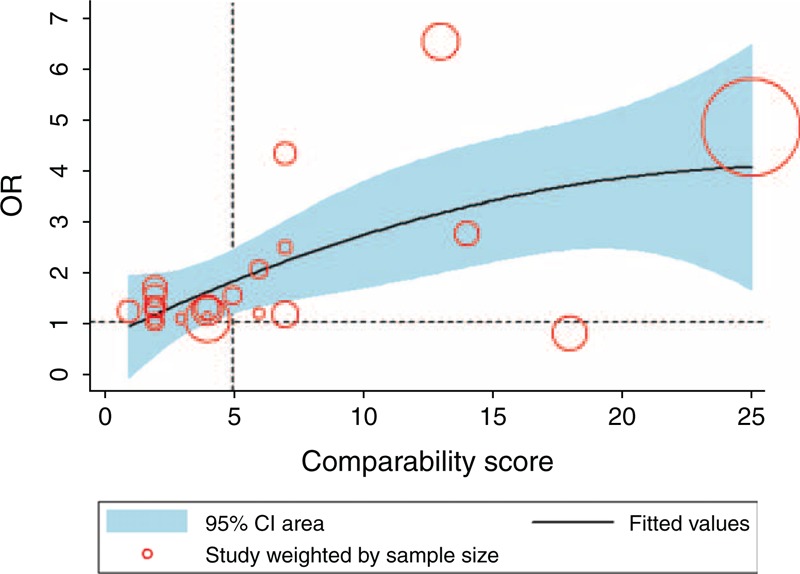
Scatter plot of the comparability scores and the ORs of the overall survival with quadratic fit and 95% CI area. CI = confidence interval.

To fix an optimal cutoff of the cumulative comparability scores, a scatter plot with quadratic fit and 95% CI area was drawn to observe the correlation between the scores and the ORs (Figure 2). A visible trend was OR increasing with a higher score. With scores higher than 5, the 95% CI area of the ORs became significantly favorable for LG.

The meta-analyses were performed based on different comparability score subsets from low to high (Supplementary Table 4 http://links.lww.com/MD/A187). There was no significant difference between the LG and OG groups, based on the subset 0–2 scores (OR = 1.22, 95% CI 0.85, 1.74, *P* = 0.29) and 3–5 scores (OR = 1.11, 95% CI 0.87, 1.41, *P* = 0.41). Based on the 6–10 and >10 score subsets, LG was superior to OG because of a more obvious imbalance between the two groups. In particular, the >10 score subset presented a significant heterogeneity (*P* < 0.01), and the random effects model was used.

**FIGURE 3:**
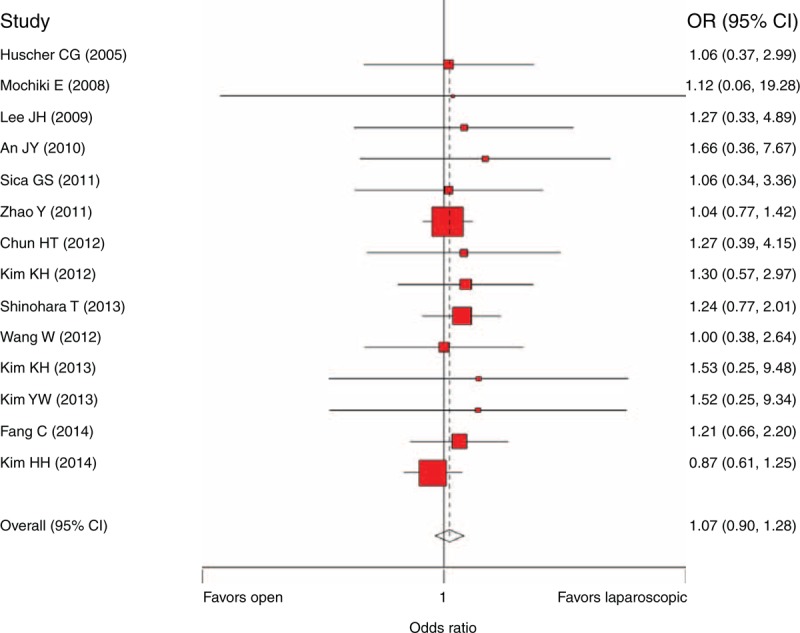
Sensitivity analysis of the 5-year overall survival comparison (including the score ≤5 studies and the matched sub-study extracted from Kim et al, 2014).^43^ CI = confidence interval, OR = odds ratio.

A sensitivity analysis was performed by repooling the comparability scores of the studies scoring 5 or lower (Figure 3). Fourteen studies were repooled, and 1807 and 1844 patients were analyzed in the LG and OG groups, respectively. The result showed that there was no longer a significant difference between the LG and OG groups (OR = 1.07, 95% CI 0.90, 1.28, *P* = 0.45), and no heterogeneity was presented (*P* = 1.00).

#### Subgroup analyses

The subgroup analyses aimed to determine whether any distinguishing subset could be benefited by LG in the long-term survival (Table 1) rates. Only the 14 studies with comparability scores of 5 or lower were included in the subgroup analyses. All of the subsets did not have a preference for LG, including the publication year, region, sample size, gastrectomy pattern, lymphadenectomy extent, amount of nodes harvested, proportion of T1–2, and proportion of N0–1. In particular, regarding lymphadenectomy as an independent confounder, the D2/D2+ only subset (OR = 1.23, 95% CI 0.91, 1.65, *P* = 0.16) and the D1/D1+ > 20% subset (OR = 0.92, 95% CI 0.66, 1.28, *P* = 0.63) had equal preference to LG or OG, if well balanced.

**TABLE 1:**
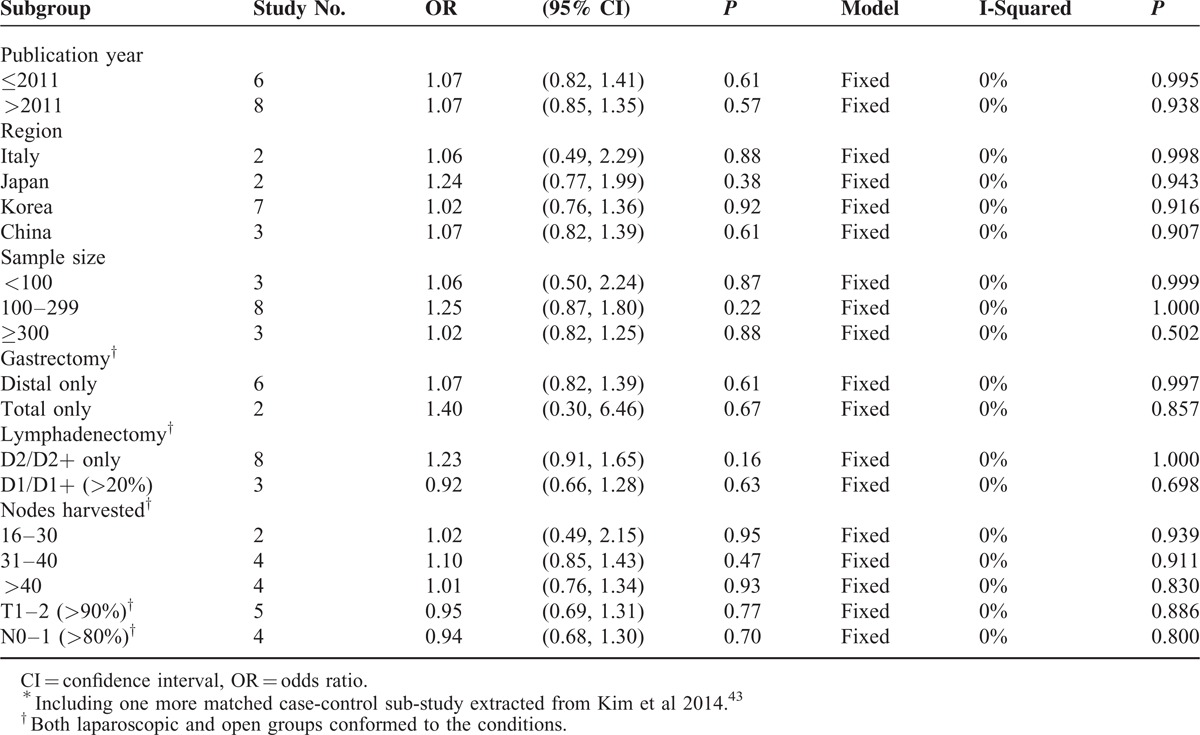
Subgroup Analyses of the 5-Year Overall Survival Based on Score ≤5 Studies^∗^

### Recurrence and Gastric Cancer–Related Death

Among those studies with comparability scores of 5 or lower, 13 studies (1172 of LG vs 1209 of OG) reported recurrence results, and six studies (578 of LG vs 544 of OG) reported gastric cancer–related deaths (Table 2). The meta-analyses showed no significant difference in the recurrence (OR = 0.83, 95% CI 0.68, 1.02, *P* = 0.08) or gastric cancer–related death (OR = 0.86, 95% CI 0.65, 1.13, *P* = 0.28) rates between the two groups. The meta-analyses based on the studies with comparability scores higher than 5 demonstrated obvious preferences for LG (Supplementary Table 4).

**TABLE 2:**
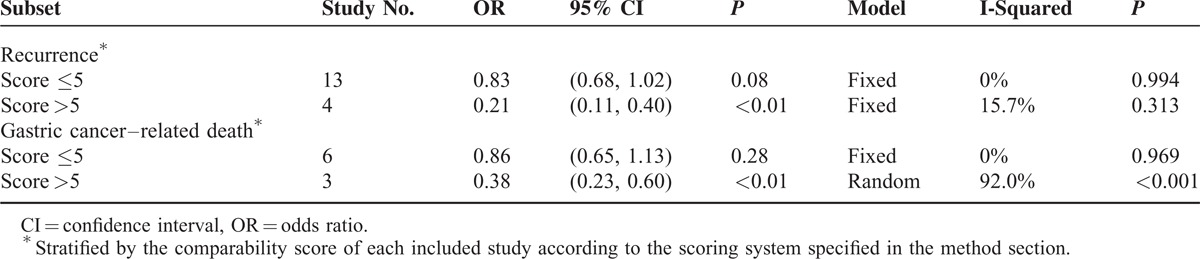
Five-Year Recurrence and Gastric Cancer–Related Death

### Stage-Specific Survival and Recurrence

The tumor stage is a determining factor in long-term survival outcomes; the stage-specific analyses were carried out on the 5-year OS and recurrence (Table 3). The proportion of the available subjects in the stage I subset was two- to threefold higher than in stage II–III. The OS and recurrence were comparable between the laparoscopic and open groups in stage I, II, or III (*P* > 0.05).

**TABLE 3:**
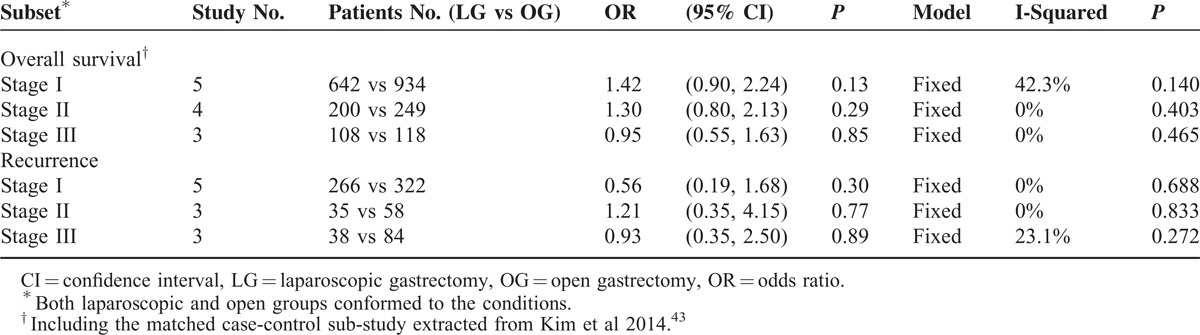
Stage-Specific Analyses of the 5-Year Overall Survival and Recurrence

## DISCUSSION

The major contributions of this systematic review compared with other meta-analyses are a comprehensive collection of available long-term survival outcomes within a much larger number of observations and a more precise consideration of confounders. The results indicate that the degree of comparability between the groups and the lymphadenectomy extent are two independent confounders that influence the estimates. Based on the well-balanced studies, the 5-year OS, recurrence, or gastric cancer–related death is comparable between LG and OG with narrow 95% CIs. Several factors such as the publication year, study region, sample size, gastrectomy pattern, lymphadenectomy extent, number of nodes harvested, and proportion of T1–2 or N0–1 do not influence the estimates, if the studies are well balanced. In particular, the stage-specific estimates obtain comparable results between the two groups.

This updated systematic review confirms the results of several previous meta-analyses. Qiu et al found a nonsignificant difference in the 3-year OS between laparoscopy-assisted and open distal gastrectomy in cases of advanced disease.^54^ Chen et al reported a similar long-term outcome between laparoscopy-assisted and open total gastrectomy.^55^ Wei et al and Ding et al found that among the patients who had undergone D2 lymphadenectomy, the OS and recurrence were comparable.^56,57^ Choi et al compared the two interventions in the advanced gastric cancer patients and found no significant difference in the long-term OS or disease-free survival.^58^ Zhang et al analyzed the early gastric cancer patients in a study from Asia and found that the recurrence rates were not different between LG andOG.^59^ Based on the randomized controlled trials, Sun et al found similar tumor recurrence rates between the laparoscopic and open groups.^60^

Compared to previously published meta-analyses, major improvement of the present meta-analysis is a full consideration of the multiple confounders for long-term survival outcome in the aspect of surgical oncology. The long-term survival outcome is influenced by many factors, including the tumor characteristics, operation pattern, and postoperative management. The comparability of these confounders contributes to the assessment of the survival estimate between LG and OG. The meta-regression and sensitivity analysis showed that the cumulative effects generated by increasing imbalance could lead to a false result favoring LG. Because of the complexity of the procedures and the uncertainty of LG, surgeons prefer to select candidates with relatively smaller tumor size and earlier stage disease for the LG group, which is the reason that the initial meta-analysis shows that LG has better 5-year OS than OG.

For the meta-analysis, we selected the well-balanced studies that make these no difference results have greater robustness in the various subsets. Although a lymphadenectomy was determined to be an independent confounder, no significant difference was shown in the extent of the dissection between the LG and OG groups, and the numbers of nodes harvested were well balanced between the two groups. In the early period, there was a shortage of lymph node dissection in LG because of a technical problem. LG was typically performed with a D1 or D1+ lymphadenectomy, and fewer nodes were harvested.^61–63^ With LG development in recent years, most surgeons are experienced in D2-LG, harvesting as many nodes as in open surgery.^33^^,64–65^ Based on current knowledge, lymphadenectomy is no longer a critical technical defect in LG.

The stage-matched meta-analyses are powerful for showing that LG is not inferior to OG for any stage of resectable disease. At the beginning, LG was only indicated in early gastric cancer patients, and the feasibility and safety of LG were widely accepted.^66^ The LG technique has recently been extended for use in advanced disease,^67^ and the controversy concerning the oncological aspects requires surgeons to pay increasing attention to the technique. This systematic review found that stage I or stage II/III diseases have comparable long-term survival and recurrence rates from LG and OG procedures. The evidence supports the use of LG in advanced resectable disease.

There are limitations to this systematic review. First, only two eligible small-sized RCTs are included in the meta-analyses, whereas the other studies are retrospective case-control studies. The quality of the original studies is an internal determining factor of evidence robustness. Because of the nature of surgical techniques, it is relatively difficult to conduct RCTs, especially double-blind studies, which are usually not feasible. There are several completed or ongoing RCTs in Japan, Korea, and China, including the JOCG-0912, JLSSG-0901, KLASS-01, KLASS-02, and CLASS-01 trials.^68–73^ These RCTs compare LG with OG in early or advanced stage disease, and the expectation of their long-term results is merited. Second, the entire observation is sufficient for the findings; however, the stage-specific analyses include only a small patient sample, especially of stage II and stage III patients. The strength of the stage-specific analyses is limited for reaching a convincing conclusion, and the trials mentioned above are required for more robust evidence. Third, the TNM staging systems have changed at different periods, which might cause systematic errors in the stage-specific analyses. A pooling analysis of the individual patient data should be a more effective method of resolving this problem. Fourth, the evidence in comparison of long-term survival outcome between LG and OG is sparse from Western countries. Therefore, the extrapolation of our findings might be limited to Western populations. Finally, although the publications seem to be clustered very close to the “no-effect” line of an OR of 1 when evaluating the funnel plot, there are 13 studies on the negative side as opposed to seven on the positive effect side (Supplementary Figure 2). It might indicate a small bias toward negative studies that are unfavorable to OG, despite of no significance in Begg test and Egger test. Thus, this is an argument why a novel and tailored measurement for quality assessment was needed to yield a more robust evidence based on homogeneous studies.^74^

The current evidence indicates that the long-term survival and recurrence rates of laparoscopic gastric cancer surgery are comparable to those of open surgery for the treatment of early or advanced stage gastric cancer, if the technical quality of the procedures is comparable. Additional high-quality RCTs are required for more confirmative conclusion.

Competing Interest: None declared.

**Authors’ Contributions:** Xin-Zu Chen and Lei Wen contributed equally to this research as co-first authors. X-Z Chen (chen_xz_wch_scu@126.com) analyzed and wrote the paper; L Wen (wenlei1998@sina.com), C-X Liu (chaoxuliu@yahoo.com), and Y-Y Rui (righton_123@163.com) searched the literature and extracted the data; and Q-C Zhao (zhaoqcfmmu@126.com), Z-G Zhou (zhou767@163.com), and J-K Hu (hujkwch@126.com) performed the quality control and proofread the paper.
